# Treatment strategies for sheep scab: An economic model of farmer behaviour

**DOI:** 10.1016/j.prevetmed.2016.12.015

**Published:** 2017-02-01

**Authors:** Emily J. Nixon, Hannah Rose Vineer, Richard Wall

**Affiliations:** Veterinary Parasitology and Ecology Group, University of Bristol, Bristol Life Sciences Building, 24 Tyndall Avenue, Bristol, BS8 1TQ, United Kingdom

**Keywords:** UK, United Kingdom, ML, macrocyclic lactone, OP, organophosphate, Sheep scab, *Psoroptes ovis*, Game theory, Farmer decision-making, Prophylaxis, Disease management

## Abstract

Ovine psoroptic mange (sheep scab) is a debilitating and damaging condition caused by a hypersensitivity reaction to the faecal material of the parasitic mite *Psoroptes ovis*. Farmers incur costs from the use of prophylactic acaricides and, if their sheep become infected, they incur the costs of therapeutic treatment plus the economic loss from reduced stock growth, lower reproductive rate, wool loss and hide damage. The unwillingness of farmers to use routine prophylactic treatment has been cited as a primary cause of the growing incidence of sheep scab in the United Kingdom (UK) since the disease was deregulated in 1992. However, if farmers behave rationally from an economic perspective, the optimum strategy that they should adopt will depend on the risk of infection and the relative costs of prophylactic versus therapeutic treatment, plus potential losses. This calculation is also complicated by the fact that the risk of infection is increased if neighbours have scab and reduced if neighbours treat prophylactically. Hence, for any farmer, the risk of infection and optimum approach to treatment is also contingent on the behaviour of neighbours, particularly when common grazing is used. Here, the relative economic costs of different prophylactic treatment strategies are calculated for upland and lowland farmers and a game theory model is used to evaluate the relative costs for a farmer and his/her neighbour under different risk scenarios. The analysis shows that prophylaxis with organophosphate (OP) dipping is a cost effective strategy, but only for upland farmers where the risk of infection is high. In all other circumstances prophylaxis is not cost effective relative to reliance on reactive (therapeutic) treatment. Hence, farmers adopting a reactive treatment policy only, are behaving in an economically rational manner. Prophylaxis and cooperation only become economically rational if the risk of scab infection is considerably higher than the current national average, or the cost of treatment is lower. Should policy makers wish to reduce the national prevalence of scab, economic incentives such as subsidising the cost of acaricides or rigorously applied financial penalties, would be required to make prophylactic treatment economically appealing to individual farmers. However, such options incur their own infrastructure and implementation costs for central government.

## Highlights

• Increased sheep scab prevalence is often blamed on UK farmers not using prophylaxis.
• A Game Theory model is developed to assess whether prophylaxis is cost-effective.
• Prophylaxis is economical with high regional prevalence and low treatment costs.
• Currently, reactive treatment is the most cost-effective strategy for most farmers.
• The implications for future policy on scab control are discussed.

## 1 Introduction

Ovine psoroptic mange (sheep scab) is a debilitating and damaging condition caused by a severe hypersensitivity reaction in sheep to the faecal material of the parasitic mite, *Psoroptes ovis* (Burgess et al., 2012). Clinical signs include dermatitis, intense pruritus and self-trauma (Berriatua et al., 2001). Sheep scab infection leads to a lower reproductive rate (Fthenakis et al., 2000), weight loss or reduced weight gain (Kirkwood, 1980, Rehbein et al., 2000a), wool loss (Rehbein et al., 2000b), additional food and acaricide costs (ADAS, 2013) higher labour costs (ADAS, 2008) and, in some cases, stock mortality (Roberts et al., 1971).

Before its deregulation in the UK, it was compulsory to treat all sheep prophylactically and by 1988 when twice yearly immersion dipping was enforced, there were fewer than 40 reported outbreaks per year (French et al., 1999). Following deregulation in 1992, many farmers abandoned prophylactic treatment, particularly with organophosphate insecticides (French et al., 1994, Bisdorff and Wall, 2008). Subsequently, the prevalence of scab increased by two orders of magnitude (Bisdorff et al., 2006, Bisdorff and Wall, 2008). Within the headline figure for national prevalence, there are significant regional variations in scab prevalence, with a study by Rose, (2011) showing 13.9% of flocks experiencing at least one outbreak per year in the uplands of Great Britain and 5.2% in the lowlands. The uplands are comprised of Scotland (average scab prevalence 7.1%), Northern England (14.1%) and Wales (20.5%) while the lowlands include Central England (3.3%), East England (5.9%) and South West England (6.4%). The regional differences in scab prevalence have been attributed to the greater use of common grazing in upland areas, since unrestricted mixing of animals facilitates transmission from infected to uninfected animals and makes prompt disease management more difficult (Rose and Wall, 2012).

The cost of sheep scab in Great Britain was estimated at £8.3 million per year (Nieuwhof and Bishop, 2005), although the true cost is likely to be higher since this estimate did not include the cost of labour, subclinical disease, or ineffective treatments. Costs are incurred because farmers are legally obliged to treat flocks visibly infected with scab with approved acaricides and from the economic loss of the reduced reproductive rate, weight, wool and skin loss of their infected livestock. Farmers also incur costs if they treat their sheep prophylactically with acaricides to prevent scab. The rapid increase in the prevalence of sheep scab in the UK following deregulation and the apparent inability to control this disease in the UK has been attributed to the fact that many farmers are unwilling to use prophylactic management given a perceived relatively low probability of infection (ADAS, 2008). As a result various sheep scab management initiatives have been launched to attempt to encourage more proactive treatment approaches (ADAS, 2008).

There are two primary prophylactic treatments for scab prevention currently licensed in the UK: a long-acting injectable formulation of the macrocyclic lactone (ML) moxidectin and the organophosphate (OP) Diazinon, used as a total-immersion plunge dip (Sargison et al., 2007). When used prophylactically, a single injection of long-acting 2% moxidectin can provide protection for up to 60 days (NOAH, 2014). Diazinon plunge dip confers protection for up to 63 days (Kirkwood and Quick, 1981). The same products can be used reactively to treat scab, as well as a range of other macrocyclic lactone products with relatively shorter periods of residual activity.

From a purely economic perspective, a farmer’s optimum strategy for scab control depends on the balance between the cost of preventative treatment (if used) and the loss in production plus the cost of reactive treatment under different risks of scab. There may also be infrastructure costs, for example dip baths, for some approaches to scab treatment. This calculation is also complicated by the fact that the risk of infection is higher if neighbours have scab and lower if neighbours treat prophylactically; having neighbours with scab has been estimated to increase the chances of scab infection by 10 times in upland flocks (Rose and Wall, 2012). If a farmer’s neighbour treats prophylactically for scab, this reduces the risk of the farmer’s flock getting scab and reduces the need to use prophylaxis. In contrast, if the neighbour’s sheep become infected with scab, the higher infection risk increases the benefits of prophylaxis. Hence, for any farmer, the risk of infection and optimum approach to treatment is contingent on the behaviour of neighbours, particularly when contact between flocks is likely, as when common grazing is used.

Farmers do not necessarily have access to information about their neighbour’s strategy or about the costs and risks of scab to aid their decision-making process. However, the use of the mathematical Game Theory approach, as conceived by von Neumann and Morgenstern (1944), allows the determination of an economic optimum strategy for a farmer based on probability, without knowledge of the neighbour’s strategy. Game Theory depicts two or more individuals (players) who will make choices that maximise their personal payoff, that is, they are rational (Myerson, 1991). The individual does not know what the other player (in this case the neighbour) will decide to do, however, the other player’s actions affect disease incidence and infection risk (Shim et al., 2012). Game Theory in a human public health context has been used to model responses to a number of infectious diseases, for example Rubella (Shim et al., 2009) and Influenza (Galvani et al., 2007). In addition, it has been applied to epidemiological studies of animal health, for example toxoplasmosis in cats (Sykes and Rychtar, 2015). The aim of this study was to use a Game Theory model to explore the relative economic costs and benefits of different strategies when making individual decisions to treat prophylactically or reactively for sheep scab.

## 2 Material and methods

### 2.1 Model construction and assumptions

A deterministic Game Theory model was constructed in Microsoft^®^ Excel (Microsoft Corporation, Redmond, WA, USA) to determine the optimum sheep scab control strategy (to treat or not treat prophylactically) for a farmer in relation to the behaviour of his/her closest neighbour. It is assumed that a farmer has only one neighbour and so the game involves two players, a farmer (known as Farmer) and his/her neighbour (Neighbour). Both players are assumed to be economically rational, that is, they are motivated solely by profit and not by any other factors. They simultaneously decide whether or not to treat their flocks prophylactically for sheep scab. Four scenarios of prophylactic treatment are possible: Farmer and Neighbour treat, Farmer treats and Neighbour does not, Neighbour treats and Farmer does not and neither treat. For all scenarios it is assumed that both farmers have the same flock size and that, if they both treat, they will use the same form of treatment. In all scenarios, both farmers apply a reactive, therapeutic treatment in the event of an infection. Every run of the model generates eight costs, one for each farmer during the four possible prophylactic treatment scenarios.

The cost to Farmer/Neighbour per year when both farmers treat prophylactically (C_tt_) is the cost of prophylaxis per ewe (and her lambs) (PC) plus the product of the probability that a farmer’s flock may get scab despite the fact that both farmers treat prophylactically (P_tt_) and the costs and losses per ewe (and her lambs) incurred if the flock does get scab (L), all multiplied by the number of ewes in the flock (N_e_).

(1) $$C_{tt}=N_{e}\cdot (PC+\left(L\cdot P_{tt}\right)$$

The cost to Farmer/Neighbour per year when they do not treat prophylactically but the other player does (C_ntt_) is the product of the probability that the flock gets scab when he does not treat prophylactically but his/her Neighbour does (P_ntt_) and costs and losses per ewe (and her lambs) incurred if the flock does get scab (L) multiplied by the number of ewes in the flock (N_e_).

(2) $$C_{ntt}=N_{e}\cdot L\cdot P_{ntt}$$

The cost to Farmer/Neighbour when they treat prophylactically but the other player does not (C_tnt_) is the prophylaxis cost per ewe (and her lambs) (PC) plus the product of the probability that a farmer’s flock will get scab when he treats prophylactically but his/her neighbour does not (P_tnt_) and costs and losses per ewe (and her lambs) incurred if the flock does get scab (L), all multiplied by the number of ewes in the flock (N_e_).

(3) $$C_{tnt}=N_{e}\cdot (PC+\left(L\cdot P_{tnt}\right)$$

The cost to Farmer/Neighbour when neither treats prophylactically (C_ntnt_) is the probability that a farmer’s flock gets scab when neither has used prophylaxis (P_ntnt_), multiplied by the costs and losses per ewe (and her lambs) incurred if the flock does get scab (L) multiplied by the number of ewes in the flock (N_e_).

(4) $$C_{ntnt}=N_{e}\cdot L\cdot P_{ntnt}$$

**Fig. 1:**
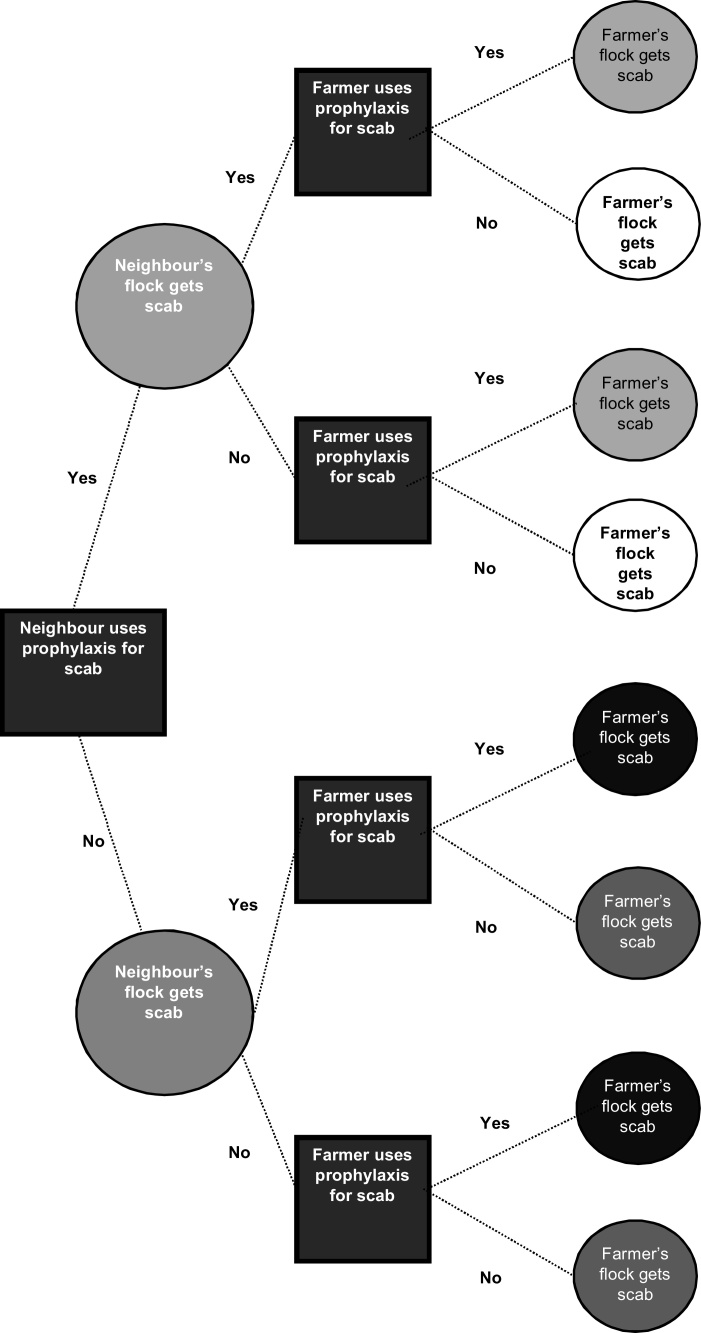
The decisions (quadrilaterals) of two farmers (Farmer and Neighbour) choosing to use or not use prophylaxis for sheep scab, and the outcomes (circles) of these decisions in a game theory model. Probability values from Table 1 were assigned to the outcomes depending on the model environment. The probability parameters of the model were then calculated as follows: *P_tt_*- sum of small light grey circles, *P_ntt_-* sum of small white circles, *P_tnt_-* sum of small black circles, *P_ntnt_-* sum of small dark grey circles. For each outcome where Farmer’s flock do get scab, subtracting the probability of this outcome from 1 will give the probability of an outcome where Farmer’s flock does not get scab.

The four probability parameters (P_tt,_ P_ntt_, P_tnt,_ P_ntnt_) were estimated based on published literature (Milne et al., 2007, Astiz et al., 2011, Rose, 2011, Rose and Wall, 2012, NOAH, 2014; Supplementary table S1). Scab transmission was considered to occur only during the autumn and winter months when clinical infections are most prevalent (French et al., 1999). A decision tree (Fig. 1) maps out outcomes resulting from whether Farmer or Neighbour treats or does not treat prophylactically and whether Farmer or Neighbour gets scab or not. The tree was used along with Table 1 to calculate the risk probabilities for upland and lowland farms when using an OP dip or ML injection for all four scenarios in the model. A full description of the probability parameter value estimations can be found in the supplementary material.

### 2.2 Costs of treatment and losses

Two treatments were considered here: an OP plunge dip with residual activity of 63 days and a long-acting injectable formulation using a macrocyclic lactone (ML) with a residual activity of 60 days. Both could be used either as a prophylactic or a therapeutic treatment of infection. There was considered to be no difference in the cost of the product when used as a prophylactic or a therapeutic treatment as the dosage will not differ in either case; product costs were obtained from veterinary wholesalers and dose rates were based on the manufacturer’s guidelines (NOAH, 2010, NOAH, 2014; Table 2). Flock costs were calculated for lowland and upland flocks based on the different lambing percentages. The costs of treatment included the cost of product and labour costs (Sewell et al., 1999, ADAS, 2013, Nix, 2016; Table 2). Dipping required the added costs of the certificate of competence (assumed to be spread over 10 years divided by the number of ewes to give a cost per ewe) plus dip disposal costs (ADAS, 2008, Myerscough College, 2014). The costs of installing and maintaining dipping facilities was not included in the calculation. At the point of treatment, the weight of all ewes was assumed to be 50 kg and lambs 30 kg (EBLEX, 2014)

Losses resulting from scab infection were calculated for lowland and upland flocks as the sum of wool productivity loss, loss in lamb sales per ewe, additional feed costs for finishing lambs per ewe, losses due to scab-induced mortality and any therapeutic treatments applied (Table 3). Extra feed costs for finishing lambs assumed that the average weight of lambs at sale was 38 kg (EBLEX, 2014). It was anticipated that infected lambs, cull ewes and rams would be treated and most would make a full recovery, hence a low mortality rate of 0.002 was assumed for infected animals. If a flock was infected then all individuals in the flock were assumed to be infected. There will be some heterogeneity in the severity of infection between individuals but the losses in wool and reproductive rate used in the model are average values and therefore the average was applied to all individuals in the flock. If a flock was not infected then it was assumed that no individual within the flock was infected and no losses would occur. Flocks could only become infected once per year.

### 2.3 Model outcomes

The model was parameterised for four environments: a lowland flock treating prophylactically with a long-acting ML injection, a lowland flock treating prophylactically with an OP dip, an upland flock treating prophylactically with a long-acting ML and an upland flock treating prophylactically with an OP dip. Within these four environments the four prophylactic treatment scenarios described in “*Model construction and assumptions”* (Eqs. (1)–(4)) were run. The output of the model gave the losses in GBP (£) for Farmer and his/her Neighbour. Each model run summed the costs for each player over a one year period.

The model identified the optimum strategy which minimised costs/losses, in each of the four prophylactic treatment scenarios (Eqs. (1)–(4)) within each environment. If the cost to Farmer when both Farmer and Neighbour treated prophylactically was greater than the cost to Farmer when only Neighbour treated prophylactically (C_tt_ > C_ntt_) then the optimum strategy was to not treat prophylactically. If the cost to Farmer when only he/she treated prophylactically was greater than the cost when neither farmer treated prophylactically (C_tnt_ > C_ntnt_) then the strategy was to not treat prophylactically, otherwise the optimum strategy was to treat prophylactically. If both C_tt_ > C_ntt_ and C_tnt_ > C_ntnt_, or C_tt^ ^_< C_ntt_ and C_tnt_ < C_ntnt_ (i.e. had the same optimum strategy), then the overall strategy was described as strictly dominant. However if two different strategies emerged (e.g. C_tt^ ^_< C_ntt_ and C_tnt^ ^_> C_ntnt_) then there was no dominant strategy. The optimum strategy for the Neighbour was calculated in the same way.

Once the optimum strategy had been found one-at-a-time (OAAT) sensitivity analyses were undertaken on three parameters to identify how variation on their values affected the optimum strategy: baseline scab risk, overall prevention cost and the cost of the prophylactic treatment product alone. Baseline risk was varied from 0 to 0.5 (0% risk of scab to 50%) at 0.005 intervals. Overall prevention cost per ewe and her lambs was varied from £0 to £2 at intervals of £0.05. The cost of the prophylactic treatment product per ewe and her lambs was also varied from 0 to £2 at intervals of £0.05.

## 3 Results

### 3.1 Farming system

The average cost per annum of having sheep scab in a lowland flock is calculated as £40.84 per ewe and her lambs (range £40.63–£41.02, Table 3) and £35.12 per ewe and her lambs in an upland flock (range £35.01–£35.38, Table 3). The minimum output per ewe (and her lambs) is normally £59.20 for lowland flocks and £48.30 for upland flocks (Nix, 2016). Prophylaxis is less expensive for upland flocks than for lowland since upland ewes have a lower lambing percentage. It is less expensive to use an OP dip for prophylaxis as opposed to injection of MLs for both upland and lowland flocks (Table 2).

**Fig. 2:**
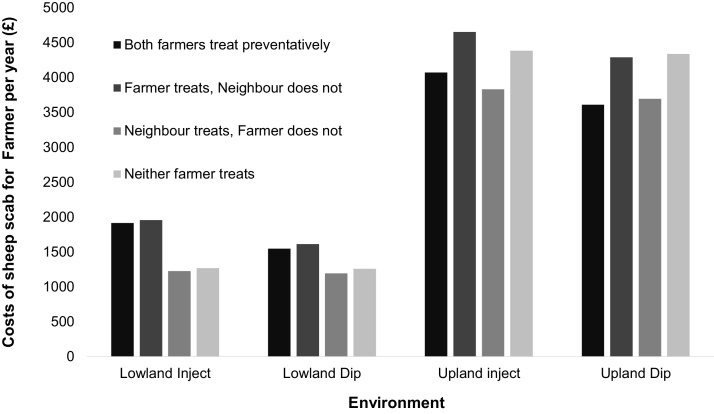
Financial losses per year (£GBP) incurred due to sheep scab and its control, by two farmers (Farmer and Neighbour) each running a 500 ewe flock, as determined by a game theory model. Farms were either upland or lowland and farmers could either treat prophylactically using a long-acting injectable ML or an organophosphate dip, or not treat. If treating, both Farmer and Neighbour would use the same product.

For lowland farmers using a long-acting ML injection prophylactically there is a strictly dominant strategy not to use prophylaxis, as it costs more for Farmer in the prophylaxis scenarios (Farmer treats prophylactically and Neighbour does not, or both treat) than in the scenarios where Farmer does not use prophylaxis (Neighbour treats and Farmer does not, or neither treats) (Fig. 2). Choosing not to treat prophylactically prevents a loss of £687 for Farmer if Neighbour treats and £688 if Neighbour does not treat. The same applies to treating prophylactically with an OP dip on a lowland farm; which costs an additional £353 per annum when Neighbour treats and £354 if Neighbour does not treat. Co-operation (both treat) using an ML would result in a loss of £644 each for Farmer and Neighbour and £289 each when using a dip.

For upland farmers who use an ML, there is also a strictly dominant strategy not to use prophylaxis (Fig. 2). Prophylaxis would cost an extra £242 per annum for Farmer if Neighbour also uses prophylaxis and £268 if Neighbour does not. If both use prophylaxis, cooperation would prevent a loss of £312 for both Farmer and Neighbour. However, the strictly dominant strategy is still to not use prophylaxis as, regardless of Neighbour’s strategy, Farmer always loses less by not treating prophylactically. In contrast, for upland farmers who treat using an OP dip, prophylaxis is a strictly dominant strategy (Fig. 2), since Farmer always loses less money overall by using prophylaxis; £84 per annum if Neighbour also uses prophylaxis and £49 if he/she does not. If Neighbour and Farmer were to cooperate (both use prophylaxis) they would each prevent a loss of £727 compared to a scenario where neither player uses prophylaxis (Fig. 2).

### 3.2 Sensitivity analysis

**Fig. 3:**
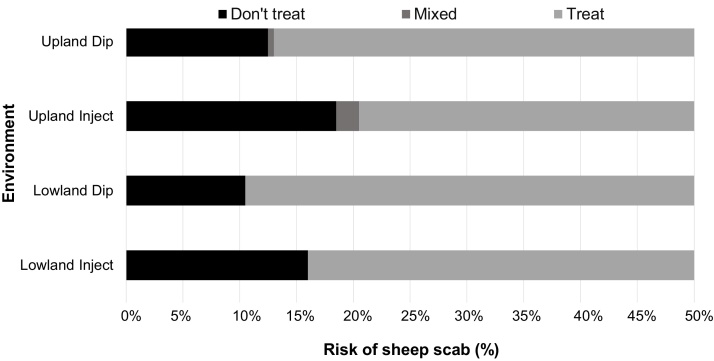
The change in dominant treatment strategy for upland or lowland flocks exposed to different risks of sheep scab as predicted by sensitivity analysis using a game theory model. Dark bar – Farmer should not use prophylaxis for scab; mid-grey − no dominant strategy (mixed); light grey – Farmer should use prophylaxis for scab.

For lowland farmers (both Farmer and Neighbour) using a long-acting ML injection there is a strictly dominant strategy to use prophylaxis only when scab prevalence is greater than or equal to 16% or to use an OP dip at a prevalence of greater than or equal to 10.5% (Fig. 3). For upland farmers using a long-acting ML injection there is a strictly dominant strategy to use prophylaxis when scab prevalence is greater than or equal to 20.5% or to use an OP dip when the prevalence greater than or equal to 13%.

**Fig. 4:**
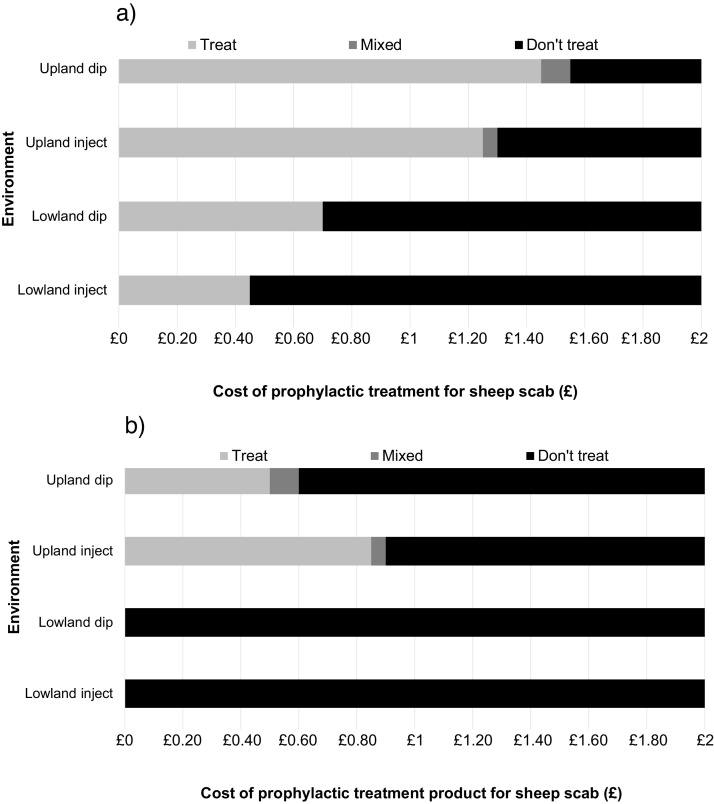
The change in dominant treatment strategy for sheep scab for upland or lowland flocks in relation to variation in (a) aggregate prevention costs and (b) cost of prophylactic treatment product only, as predicted by sensitivity analysis using a game theory model. Dark bar – Farmer should not use prophylaxis for sheep scab; mid-grey – no dominant strategy (mixed); light grey – Farmer should use prophylaxis for sheep scab.

For lowland farmers, the strictly dominant strategy not to use prophylaxis is unaffected by the cost of the product at the range of treatment costs examined (Fig. 4b). However, reducing the overall prevention cost (treatment plus labour) did make it economically viable to use prophylaxis in the lowlands when this was equal to or below £0.70 (dipping) or £0.45 (injecting) (Fig. 4a). For upland farmers, varying the treatment product cost alone was enough to change the strategy, and a strategy of prophylaxis became strictly dominant when product costs were less than or equal to £0.50 (dip) or £0.85 (inject) per ewe and her lambs (Fig. 4b).

## 4 Discussion

Game Theory recommends or explains decisions of individuals that are affected by and have implications for the decisions of others (Nash, 1951). It has been used previously to inform disease management, for example in salmon farming (Murray, 2014) and in the use of antibiotics (Porco et al., 2012). The Game Theory model developed here, based on data available in the literature, has been used to identify optimum economic strategies (to treat or not treat prophylactically for scab) in relation to the unknown strategy of a neighbouring sheep farmer. The model developed utilises all available data on the control and disease costs of scab, taking into account factors such as extra finishing costs and lower reproductive rates which have not always been included in previous estimates (Scott et al., 2007).

Not applying prophylactic treatment is a strictly dominant strategy if treating prophylactically with an ML injection on upland farms or with a ML or OP dip on lowland farms. This is because prophylactic treatment costs are high relative to the risks of infection. The only situation where prophylactic treatment was a strictly dominant strategy was for upland farmers using OP dip. However, the savings farmers might make compared with a scenario of no prophylactic treatment through prophylaxis are low (£84 per year or £49 per year depending on whether the neighbour does or does not use prophylaxis) and therefore in practice upland farmers may still choose not to use prophylaxis. It should also be noted that the costs of dipping infrastructure were not included in the calculation presented here and for farmers where such facilities were unavailable, the capital costs needed for their construction would again make prophylactic dipping uneconomic.

Cooperation (both farmers using prophylaxis) in upland farms always results in a lower mean loss per farmer (Fig. 2). However, from a Game Theory perspective, there is still a strictly dominant strategy to not treat prophylactically if the treatment is by injection. This situation emulates the most common Game Theory example, the Prisoner’s Dilemma (Axelrod and Hamilton, 1981). In both games, no players have an incentive to deviate from their strategy of non-cooperation and so, if we assume rationality, they will never choose to cooperate. It has already been suggested that a lack of compliance by certain farmers during the compulsory dipping period of 1972–1992 was a key reason for the failure to eradicate sheep scab during this time and for its subsequent spread (Rose, 2011). The findings reported here suggest that non-cooperation is an economically rational response, as also suggested by Milne et al. (2007). Hence, if future control programs require compliance by all farmers, economic incentives or penalties would be required to encourage farmers to deviate from their most economically rational strategy.

Cooperation was still less favoured in lowland farms under current scab prevalence since there was a strictly dominant strategy not to use prophylaxis. In fact, if both Farmer and Neighbour co-operated in treating prophylactically the mean loss would be greater than if neither cooperated (Fig. 2). Existing economic data also supports the idea that it is not always economically viable to use prophylaxis. For example, in Scotland in 2006, £5.1 million was spent on prophylaxis while losses due to scab were estimated to cost only £0.6 million (ADAS, 2008) (although the estimate of losses did not take into account all the costs, for example reproductive losses).

Although, given the most current average national prevalence values for scab (13.9% upland and 5.2% lowland, Rose, 2011), the current strictly dominant strategy is to only use prophylaxis if using an OP dip on an upland farm, the sensitivity analysis demonstrates that as the risk of scab increases this strategy changes. The prophylactic use of ML injections on upland farms becomes a strictly dominant strategy when the prevalence is above or equal to 20.5% and in lowland farms when the prevalence is above or equal to 16% (Fig. 3). Dipping on lowland farms becomes strictly dominant when the prevalence is above 10.5% (Fig. 3), with the difference between treatment types being attributed to lower cost of dipping based on a flock of 500 ewes. These results suggest that higher prevalence regional hotspots, with higher than average prevalence, could be good targets for prophylaxis programs. For example, in Wales, the prevalence has been reported to be above average at 20.5% (Rose, 2011) and at 35% (Cross et al., 2010) and therefore not only dipping, but also injecting with a long-acting ML at current costs-per-dose (Table 2) would be a cost effective strategy in controlling sheep scab. If other higher prevalence regional hotspots can be identified (in either uplands or lowlands) prophylaxis might also be an optimum strategy in these areas. Although, of course such targeted programmes bring with them additional management, surveillance and infrastructure costs that must be borne by central government of distributed between individual farmers in the area.

The prevalence estimates used in our study were based on a survey of around 400 sheep farmers in 2008 (Rose, 2011) and were found to be similar to those from a previous survey (Bisdorff et al., 2006). Although not completely up to date, these prevalence figures give a good representation of what current scab risks may be in different regions in Great Britain. Unfortunately, they are only able to give prevalence estimates at a relatively crude regional scale which limits the identification of hotspots, although spatial models of the distribution of reported scab outbreaks may aid the identification of particularly high risk regions (Rose et al., 2009). Furthermore, prevalence can be underestimated since farmers are often reluctant to admit to the presence of scab in their flocks (Cross et al., 2010) or may not report outbreaks if scab is a persistent problem within their flock or area. However, this could be overcome by the use of the Randomised Response Technique, a method which protects the farmers’ anonymity and appeared to result in higher estimates of prevalence when employed in a survey by Cross et al. (2010) than found in previous surveys. In order to collect continuous prevalence data, media reporting methods such as the use of mobile applications could be used, as discussed by Walker (2013). More detailed data on scab prevalence in certain regions would enable our model to inform farmers more accurately on whether and how they should be treating.

The sensitivity analysis of the cost of the prophylactic treatment product demonstrated that subsidising this cost alone was not enough to incentivise lowland farmers to use prophylaxis (Fig. 4b) and that the overall prevention cost (product cost plus labour costs, dip disposal costs etc., see Table 2) would need to be less than or equal to £0.70 (dip) or £0.45 (inject) per ewe (+ lambs) for prophylaxis to be economically viable for lowland farmers (Fig. 4a), based on projections for ewe output for 2016 (Nix, 2016). Although dipping may be economically viable in the uplands without subsidy, there have been concerns relating to its potential harmful effects to the environment and the operator, which may prevent certain farmers from choosing this method of treatment (Sargison et al., 2007). Subsidising the cost of ML product per ewe (+ lambs) to £0.85 or less would make it economically viable for upland farmers to treat with injectable MLs as an alternative (Fig. 4b), based on projections for ewe output for 2016 (Nix, 2016). Alternatively, rigorously applied financial penalties would have the same economic effect.

Whether government would subsidise or otherwise incentivise preventative treatment enters the realm of balancing political against economic imperatives: clearly centralised management would bring a range of associated costs. These would include start-up, fixed or overhead costs (Tisdell, 2009) which could include further research costs, costs for contract negotiations, disease surveillance costs and costs relating to the monitoring of compliance and uptake (Rushton and Leonard, 2009). All of these factors would need to be considered in a cost-benefit analysis as described by Tisdell (2009) before instigation of such a program. There has been debate in recent years as to whether animal health should be seen as a public or a private good and consequently whether the government should have a role in providing this service (Rushton and Leonard, 2009).

One significant problem with the modelling approach used here is that it assumes that farmers are strictly rational decision makers driven by economic concerns. In reality, however, the control of disease takes place within the entire-farm context and farmers have other goals, values and influences which also affect their decision-making processes, such as job satisfaction, peer pressure, animal welfare, farm succession, maintaining a way of life, stressful circumstances, personality and attitude to risk (Wallace and Moss, 2002, Long, 2013). The model presented here considers a Farmer and his/her Neighbour, each with a flock of 500 ewes. The costs of prophylactic and therapeutic treatment will vary according to flock size (with economies of scale), and therefore the point at which prophylactic treatment becomes economically viable may vary with flock size and predicted ewe output (e.g. Nix, 2016) in addition to the factors explored in the sensitivity analyses. A further limitation of this model is that it can only simulate a scenario with a single neighbour when in reality, farmers often have multiple neighbours. An extension of the model might consider the impact of group cooperation and how this dynamic would change the optimum strategy for the farmer; nevertheless the current single-neighbour scenario is a useful first step in this approach. A number of studies looking at spatial prisoner’s dilemma games have concluded that spatial structure encourages cooperation (Nowak and May, 1992, Hubermann and Glance, 1993, Nowak, 1993, Nowak et al., 1994, Killingback et al., 1999). When co-operators form clusters, the benefits that come from mutual cooperation make them successful even when exploited by defectors along the cluster boundaries (Hauert and Doebeli, 2004). Lindgren and Nordahl (1994) have also shown that unconditional co-operators do much better in spatial prisoner dilemma games than in non-spatial. A different result might come from a model which looks at the problem spatially. This might be a good basis for coordinating a cooperative community treating program for clustered farms in the UK.

## 5 Conclusions

The model outputs have shown that, given current scab prevalence and sheep scab treatment costs, prophylaxis employing OP may only be economically viable in upland farms (long-acting ML injections may also be cost effective in high prevalence regions such as Wales). Using prophylaxis in lowland farms is not cost effective. However, identifying higher prevalence regional hotspots that could be good targets for economically viable prophylaxis programs may be a productive approach. Only subsidising the overall cost of prevention would incentivise lowland farmers to use prophylaxis, assuming treatment choices are economically rational. The costs associated with sheep scab control and treatment have been estimated for both upland and lowland farms and together with this model provide a useful insight into the underlying drivers informing management decisions by farmers and may help in policy formulation.

## Conflicts of interest

None.

## Figures and Tables

**Table 1:** Probability of possible outcomes in a game theory model where a farmer and his/her neighbour are deciding whether to use prophylaxis for sheep scab.

Probability	Dip		Inject	
	Upland	Lowland	Upland	Lowland
Pr(scab | treatment)	0.092	0.036	0.099	0.041
Pr(no scab | treatment)	0.908	0.964	0.901	0.959
Pr(scab | no treatment)	0.139	0.052	0.139	0.052
Pr(no scab | no treatment)	0.861	0.948	0.861	0.948
Pr(infection | neighbour infected)	0.91	0.2	0.91	0.2
Pr(healthy | neighbour infected)	0.09	0.8	0.09	0.8

These values were estimated using data from the literature (see supplementary material) and were used along with Fig. 1 in order to estimate the values of the probability parameters in the model.

**Table 2:** Estimation of prevention costs for sheep scab by injection of a long-acting macrocyclic lactone or an organophosphate dip.

Costs of prevention	Lowland	Upland	
Injecting	Cost (£)	Cost (£)	Sources
Cost of long-acting ML injection per ewe (+ lambs)	£1.42	£1.37	NOAH (2014)
Labour per ewe (+ lambs)	£0.40	£0.40	ADAS (2013)
Total cost of injecting per ewe (+ lambs)	£1.82	£1.77	–
Dipping	–	–	–
Cost of OP dipping product per ewe (+ lambs)	£0.39	£0.39	NOAH (2010)
Labour per ewe (+ lambs)	£0.86	£0.83	Sewell et al., 1999, Nix, 2016
Cost of certificate of competence per ewe (+ lambs)	£0.01	£0.01	Myerscough College (2014)
Dip disposal costs per ewe (+ lambs)	£0.10	£0.11	Appendix 3, ADAS (2008)
Total cost of dipping per ewe (+ lambs)	£1.36	£1.34	–

Cost of certificate of competence is a one −off payment assumed to be valid for 10 years.

**Table 3:** Costs of sheep scab infestation for lowland and upland farms in the UK.

Costs of sheep scab	No scab		Scab		Losses due to scab		Source
	Lowland	Upland	Lowland	Upland	Lowland	Upland	
Wool sales per ewe	£2.40	£1.90	£1.57	£1.24	£0.83	£0.66	Nix (2016); Rehbein et al. (2000b)
Lambing ratio	1.7	1.6	1.29	1.2	n/a	n/a	Fthenakis et al. (2000); Nix (2016)
Lamb sales per ewe	£114.72	£99.20	£86.04	£74.40	£28.68	£24.80	Fthenakis et al. (2000); Nix (2016)
Finishing food costs:	–	–	–	–	–	–	EBLEX (2014); Hindson (2002); Kirkwood (1980); NADIS (2015); Rehbein et al. (2000a)
- per lamb	£25.09	£23.01	£32.58	£29.88	£7.49	£6.79	
- for lambs per scabby ewe	–	–	–	–	£9.67	£8.15	
Lamb mortality costs per ewe	£0	£0	£0.17	£0.15	£0.17	£0.15	Nix (2016)
Cull ewe and ram mortality costs	£0	£0	£0.03	£0.02	£0.03	£0.02	Nix (2016)
Treatment:	–	–	–	–	–	–	Table 2
- injection per scabby ewe (+ lambs)	£0	£0	£1.82	£1.77	£1.64	£1.60	
- dip per ewe (+ lambs)	£0	£0	£1.37	£1.34	£1.25	£1.23	
Total loss per ewe (+ lambs)	–	–	–	–	–	–	
Injecting	–	–	–	–	**£41.02**	**£35.38**	
Dipping	–	–	–	–	**£40.63**	**£35.01**	

Per ewe (+ lambs) refers to costs for the ewe plus the costs for its lambs produced in a one year period (determined by the lambing ratio).
