# Supplementary material for: Treatment strategies for sheep scab: An economic model of farmer behaviour
Source: Prev Vet Med. 2017 Feb 1;137(Pt A):43–51. doi: 10.1016/j.prevetmed.2016.12.015 (PMC5292105; doi:10.1016/j.prevetmed.2016.12.015)
Supplement: Supplementary file 1 [file mmc1.docx]

**Supplementary material**

This material explains in more detail the probability calculations which lead to the results shown in Table 1. References used here are found in full in the bibliography of the main body of text.

*Probability of getting scab when using prophylaxis*

The probability that a farmer’s flock gets scab during the autumn and winter months when prophylactically treating (Pr(S|T) was calculated using the following equation:

| $Pr(S\vert T)=\left( Y\cdot N\cdot R_{i} \right)+((1-Y\cdot N)\cdot R)$ | (1s) |
| --- | --- |

Where *Y* is the proportion of the autumn and winter months protected, *N* is the number of times the treatment is applied, *R_i_* is the risk of scab when prophylactic treatment is used and *R* is the baseline risk where farmers treat reactively (i.e. in response to an infestation) and forego prophylactic treatment.

The protection conferred by prophylactic treatment is transitory. Therefore, this equation takes into account the risk of scab when a flock are protected by the prophylactic treatment ($Y\cdot N\cdot R_{i}$) and the risk of scab during the rest of the autumn and winter when the prophylactic treatment is no longer having an effect$((1-Y\cdot N)\cdot R$), giving an overall risk of scab for this time period.

Table S1: Parameters and the data sources used to calculate their values in equations 2s-5s of a game theory model depicting the financial outcomes for two neighbouring farmers deciding whether or not to use prophylaxis against sheep scab. Corresponds with Table 1.

| **Probabilities** | **Shorthand** | **Sources and calculations** |
| --- | --- | --- |
| Pr(scab \| treatment) | Pr(S\|T) | Equation (1s), using the assumed probability of losses being prevented by application of organophosphate dip (99.5%, Table 20, Milne (2007)) and residual activity (63 days, Kirkwood and Quick 1981). Also, the residual activity of 1 injection of Cydectin 2% LA (60 days, NOAH 2014) and efficacy (98.1% calculated efficacy at 54 days (closest to 60), in Table 3 (incidence), Astiz *et al*. 2011).  Baseline risk was the same as the prevalence (Pr(scab \| no treatment) |
| Pr(no scab \| treatment) | Pr(NS\|T) | 1- Pr(scab \| treatment) |
| Pr(scab \| no treatment) | Pr(S\|NT) | Upland prevalence was the average prevalence of Scotland, Northern England and Wales. Lowland was average of Central, East and South West England (Rose, 2011) |
| Pr(no scab \| no treatment) | Pr(NS\|NT) | 1-Pr(scab \| no Treatment) |
| Pr(infection \| neighbour infected) | Pr(I\|NI) | Calculated using the odds ratio for neighbours with scab from Table 2, Rose and Wall (2012) for upland (Probability= odds ratio/ (1+odds ratio)).  Estimated for lowland based on evidence from Rose and Wall (2012) |
| Pr(healthy\| neighbour infected) | Pr(H\|NI) | 1-Pr(infection \| neighbour infected) |

*Model probability parameters*

The four probability parameters from the main body of text are the probability of getting scab when:

- Farmer and neighbour use prophylaxis (P_tt_)
- Farmer does not use prophylaxis, Neighbour does (P_ntt_)
- Farmer uses prophylaxis, neighbour does not (P_tnt_)
- Neither player uses prophylaxis (P_ntnt_)

These were calculated using the following equations, the decision tree in the main body of text (Fig 1) and Table S1 (which describes the extra parameters). The parameters in bold refer to those probabilities that relate only to the neighbour’s decision.

| P_tt_= sum of small light grey circles in Fig 1  $P_{tt}=\boldsymbol{Pr}\left( \boldsymbol{S\vert T} \right)\cdot\left( \left( Pr\left( S\vert T \right)+Pr\left( NS\vert T \right)\cdot\Pr\left( I\vert NI \right) \right)+Pr\left( S\vert T \right) \right)$ | (2s) |
| --- | --- |
| P_ntt_= sum of small white circles in Fig 1  $P_{ntt}=\boldsymbol{Pr}\left( \boldsymbol{S\vert T} \right)\cdot\left( \left( Pr\left( S\vert NT \right)+Pr\left( NS\vert NT \right)\cdot Pr\left( I\vert NI \right) \right)+Pr\left( S\vert NT \right) \right)$ | (3s) |
| P_tnt_= sum of small black circles in Fig 1  $P_{tnt}=\mathbf{Pr}\left( \boldsymbol{S\vert NT} \right)\cdot\left( \left( Pr\left( S\vert T \right)+Pr\left( NS\vert T \right)\cdot Pr\left( I\vert NI \right) \right)+Pr\left( S\vert T \right) \right)$ | (4s) |
| P_ntnt_= sum of small dark grey circles in Fig 1  $P_{ntnt}=\boldsymbol{Pr}\left( \boldsymbol{S\vert NT} \right)\cdot\left( \left( Pr\left( S\vert NT \right)+Pr\left( NS\vert NT \right)\cdot Pr\left( I\vert NI \right) \right)+Pr\left( S\vert NT \right) \right)$ | (5s) |
